# Self-Medication: Attitudes and Behaviors Among Pharmacy and Medical Students

**DOI:** 10.3390/pharmacy13050127

**Published:** 2025-09-04

**Authors:** George Jîtcă, Carmen-Maria Jîtcă, Mădălina-Georgiana Buț, Camil-Eugen Vari

**Affiliations:** 1 Department of Pharmacology and Clinical Pharmacy, Faculty of Pharmacy, George Emil Palade University of Medicine, Pharmacy, Science and Technology of Târgu Mureș, 540139 Târgu Mureș, Romania; camil.vari@umfst.ro; 2 Doctoral School of Medicine and Pharmacy, Institution Organizing Doctor’s Degree University Studies, George Emil Palade University of Medicine, Pharmacy, Science and Technology of Târgu Mureș, 540139 Targu Mures, Romania; carmenrusz20@gmail.com (C.-M.J.); madalina-georgiana.batrinu@umfst.ro (M.-G.B.); 3 Department of Biochemistry, Faculty of Pharmacy, George Emil Palade University of Medicine, Pharmacy, Science and Technology of Târgu Mures, 540139 Târgu Mureș, Romania

**Keywords:** self-medication, pharmacy, medicine, students, university

## Abstract

Self-medication is increasingly prevalent among healthcare students, raising concerns about the adequacy of current medical education in promoting safe medication practices. This study aimed to assess the frequency, motivations, and perceptions of self-medication among medical and pharmacy students and to identify educational gaps. A cross-sectional survey was conducted using a structured, anonymous questionnaire distributed to medical and pharmacy students at a single academic institution. The questionnaire assessed self-medication frequency, substances used, motivations, perceived risks, confidence in knowledge, sources of information, and attitudes toward curriculum improvements. Over 50% of participants reported practicing self-medication at least once a month. The most commonly used substances were analgesics and dietary supplements. Main motivations included recognition of symptoms, confidence in personal knowledge, and avoidance of waiting times. Despite receiving university instruction on self-medication risks, students continued to self-medicate, with many relying on the internet as a primary source of information. Only 8% felt very confident in counseling patients on self-medication. A majority (over 70%) expressed a strong interest in integrating dedicated educational modules into the curriculum. There is a clear need for improved, practice-oriented education on self-medication. Future interventions should focus on interdisciplinary teaching, digital literacy, and simulation-based training to foster safer medication practices.

## 1. Introduction

Self-medication, including the use of over-the-counter (OTC) drugs, is a common practice worldwide. While often appropriate for minor and easily recognizable symptoms, it requires careful consideration, proper knowledge, and awareness of potential risks. This study aimed to explore self-medication behaviors, confidence levels, and sources of information among medical and pharmacy students, without assuming that all self-medication is inappropriate. This practice is becoming more widespread, not only among the general public but also among healthcare students. Even with a solid background in pharmacology and pharmacotherapy, students may engage in self-medication, making it crucial to emphasize responsible practice, as they can serve as role models for patients and the wider community [1,2,3,4,5].

Research at the international level has revealed a significant prevalence of self-medication among pharmacy students. For instance, a study carried out in India found that 86% of students had practiced self-medication, with 70% using drugs based on suggestions from friends [6]. Another study conducted in Iran revealed that 44.8% of university students reported using non-prescription medications, with analgesics and antibiotics being the most frequently used [7]. In Romania, a study carried out in the northeastern region found that 41% of students had taken at least one antibiotic in the previous six months, with 44% of them doing so without consulting a physician [7]. These data suggest a frequent practice of self-medication among Romanian students, including those studying pharmacy. Recently, the law in Romania regarding the dispensing of antibiotics was amended under Order No. 63/2024, as a response to the widespread misuse and the ease with which these drugs were previously obtained from pharmacies.

The motivations for engaging in self-medication are varied and include the desire to save time and money, previous experience with the same medications, and the perception that the condition is not serious. Additionally, easy access to OTC medications and social influences can contribute to this practice [6,8]. Although medical students possess theoretical knowledge about proper medication use, their self-medication behaviors are influenced by factors such as lifestyle, academic pressure, and easy access to medications. While self-medication can be appropriate for minor symptoms, insufficient caution or misuse may occasionally lead to adverse effects or drug interactions [8,9,10,11,12].

Self-medication is not something that is only found among students with a specific medical profile, but also in the general population, both in developed and developing countries [13]. It should be emphasized that self-medication is not inherently wrong, rather, its appropriateness depends on the context. Self-medication is a component of self-care, a common practice that, when conducted responsibly, can be beneficial for managing minor symptoms. The World Health Organization (WHO) defines self-care as the ability of individuals, families, and communities to promote health, prevent disease, and manage illness with or without the support of a health professional. Within this broader concept, self-medication refers to the use of approved medicines, particularly OTC products, by individuals to treat perceived symptoms or conditions. The WHO emphasizes the importance of responsible self-medication, which requires access to accurate information and the appropriate use of medicines, in order to maximize benefits and minimize potential risks [14]. The use of leftover medications from previous treatments is also considered a form of self-medication [15,16]. However, irrational use may reduce treatment effectiveness, increase the risk of adverse effects, tolerance, dependence, or toxicity due to overdose, mask underlying diagnoses, or lead to drug interactions if concurrent treatments are prescribed [17,18]. Additional risks include unintentional duplication of active substances found in products with different names, inappropriate routes of administration, or improper storage [16]. While irrational self-medication may contribute to increased healthcare costs or, in severe cases, hospitalization, it is important to note that responsible use is generally safe and effective. Media advertising of medicines and supplements can also influence individuals to engage in self-medication [19,20].

The aim of this article is to analyze the prevalence and factors influencing self-medication among medical students, highlighting both the theoretical knowledge that should prevent risky behaviors and the external aspects that may contribute to such choices. The study now clearly focuses on examining the prevalence of self-medication among students who have completed the pharmacology course, exploring the relationship between their pharmacological knowledge, confidence in that knowledge, and self-medication behaviors, as well as identifying the types of medications most frequently used and the potential risks associated with these practices. Furthermore, the potential impact of self-medication on health will be discussed, as well as the responsibility these students should assume as future healthcare professionals.

## 2. Materials and Methods

### 2.1. Study Design, Setting, and Participants

A quantitative, cross-sectional study was conducted between May and June 2025, targeting students enrolled in the Medicine and Pharmacy programs at the George Emil Palade University of Medicine, Pharmacy, Science and Technology of Târgu Mureș, Romania. The aim of the study was to evaluate the behavior and attitudes toward self-medication among students in these programs, specifically those in the 3rd year of Medicine and the 4th year of Pharmacy. The sampling was random. The questionnaire targeted only 3rd-year medical and 4th-year pharmacy students, as they had completed pharmacology courses covering the main drug classes. This criterion was essential to ensure respondents had the minimum knowledge needed to understand drug mechanisms, indications, contraindications, and risks. Including students without such training could have led to superficial answers and biased data. Focusing on these study years provided a comparable framework to analyze the relationship between pharmacological knowledge, self-medication behaviors, and risk perception.

Out of a total student population of approximately 500, 224 online questionnaires and paper questionnaires were completed. The study included participants of both genders, and the sample size was calculated using the Raosoft software (http://www.raosoft.com/, accessed on 25 July 2025) available on the developer’s website. The required sample size was determined based on a 95% confidence level, an assumed response distribution of 50%, and a ± 5% margin of error.

### 2.2. Questionnaire

A 16-item questionnaire was designed to evaluate students’ attitudes, behaviors, and perceptions toward self-medication, as well as to identify the factors that contribute to this practice. The questionnaire was structured into five sections. The first section collected sociodemographic data (3 items). The second section explored self-medication behaviors, specifically the frequency and types of self-medication practiced (3 items). The third section assessed students’ confidence in their acquired knowledge (2 items). The fourth section examined beliefs and perceptions about the safety of self-medication (4 items). The fifth section aimed to identify sources of information and gather data on exploratory factors and aspects related to professional education (4 items). The content validity of the questionnaire was evaluated by a group of experts from our faculty. Additionally, a pilot study was conducted to assess the clarity of the questions and to make necessary modifications based on the feedback received from this test. The questionnaire was distributed both via the Google Forms platform and in paper format, with all students participating voluntarily and anonymously in the study. Where applicable, the questionnaire allowed only a single response to capture the most relevant category of medication or source of information during the reference period. This design aimed to identify the primary self-medication pattern at the individual level and to reduce potential overlap. All types of medications were considered, including OTC drugs, prescription-only medicines used without a prescription, and vitamins/supplements. The questionnaire is available as a Supplementary Material.

### 2.3. Statistical Analysis

Data were exported and processed using Microsoft Excel and analyzed with GraphPad Prism Software version 9 (San Diego, CA, USA). Descriptive statistics, presented as percentages, were used to determine the frequency distributions of variables such as gender, study year, age, self-medication frequency, types of medications used, sources of information, acceptance of self-medication, and perception of risks. Central tendency (mean) and variability (standard deviation) were calculated for confidence in pharmacological knowledge and self-perceived readiness for patient counseling. Normality was tested using the Kolmogorov–Smirnov test. Statistical tests including Chi-square or Fisher’s exact test (associations between gender and type of self-medication, gender and frequency of self-medication, and confidence in knowledge and sources of information), *t*-test or Mann–Whitney U test (comparisons of confidence levels between females and males, comparisons of confidence levels by specialization, comparisons of self-perceived readiness to counsel patients), and Spearman’s correlation (associations between confidence in own knowledge and frequency of self-medication, and between confidence level and self-perceived readiness to counsel patients about self-medication risks) were applied as appropriate. *p* < 0.05 was considered statistically significant. The internal consistency of the scale assessing perceived pharmacological competence and self-perceived readiness to counsel patients was evaluated using two relevant items. The Pearson correlation between them was r = 0.44, corresponding to a Cronbach’s alpha of approximately 0.61, indicating low to moderate internal consistency. Given the small number of items, the results should be interpreted with caution.

## 3. Results

No questionnaires were excluded, as all met the inclusion criteria of students enrolled in the Medicine and Pharmacy programs in the 3rd and 4th years.

### 3.1. Sociodemographic Data

Of the 224 students included in the study, 71.4% were female. The average age was 22 years (range: 20–37), and most students were enrolled in the Medicine program, as seen in Table 1.

### 3.2. Frequency and Type of Self-Medication

Among the 224 students, self-medication was most commonly reported every few months (44.6%), followed by monthly (32.6%) and weekly (22.3%), as seen in Table 2. Analgesics/antipyretics and vitamins/supplements for cognitive support were the most frequently used, with higher usage among females. Students were informed that both vitamins (as multivitamin complexes) and supplements (e.g., lecithin, Ginkgo biloba) were considered in the context of products used to support learning and cognitive performance. The main reasons included mild symptoms and confidence in pharmacology knowledge. Therefore, an association between gender and the type of self-medication was observed, with females reporting it more frequently; this may be partly due to the relatively small number of male participants (χ^2^ = 6.757, *p* = 0.034). No significant associations were found for frequency or between confidence and self-medication (Spearman’s r = −0.041, *p* = 0.545).

### 3.3. Confidence in Their Own Knowledge

Most respondents reported being fairly or very confident in their knowledge of self-medication. Around two-thirds indicated that the risks of self-medication were covered during their courses. No statistically significant difference in confidence was found between females and males (*p* = 0.0723), although females showed a slight tendency toward higher confidence. Confidence levels were also compared by specialization, with no significant differences found (*p* = 0.3707).

### 3.4. Perception About Self-Medication

Most participants considered self-medication acceptable, either context-dependently or in general, and perceived a range of potential risks, including drug interactions, adverse reactions, and misdiagnosis, as seen in Table 3. The vast majority reported no negative experiences, while a few noted mild adverse effects, such as palpitations, allergic reactions, or gastritis.

### 3.5. Eploratory Elements and Professional Training

Most students relied on the internet and books/lectures for self-medication information, with analgesics and anti-inflammatories used most frequently, followed by dietary supplements for studying or relaxation, as seen in Table 4. Students emphasized the need for educational campaigns and reliable sources, including potential apps or 24/7 services. About 71% supported including self-medication in the curriculum. A comparison between study programs regarding self-perceived readiness for patient counseling revealed no significant differences (*p* = 0.1392). Confidence in counseling patients varied, with nearly half feeling prepared, and confidence positively correlated with self-perceived readiness to advise on self-medication risks (r = 0.44, *p* < 0.001). Additionally, an analysis comparing the two study programs among students who rated themselves as fairly or very confident in their knowledge and sources of information showed a significant association, χ^2^ (12.29, 4, *p* < 0.0154).

## 4. Discussion

The results of this study align with previous reports showing a high prevalence of self-medication among medical and pharmacy students, with over 50% using medications at least monthly. In Romania, many radio and television ads promote OTC medications and dietary supplements, which are regulated under Law no. 95/2006 and subsequent ANMDM norms [21]. While advertising prescription-only medicines to the public is prohibited and all promotional materials must be approved by ANMDM [22,23], social media presents challenges in monitoring content, targeting, and compliance. Influencer posts and sponsored content can bypass oversight, potentially encouraging self-medication or creating misleading perceptions of safety. Stricter regulation and adaptation to digital platforms are therefore necessary [24]. Recent studies have highlighted the increasing presence of pharmaceutical advertising on social media platforms, raising important questions about content accuracy and regulatory compliance [25,26,27,28].

Previous studies have reported high rates of self-medication among students. For example, over 50% of respondents in Iran used self-medication, with antibiotics being used much more frequently than in our study (74.4% vs. 5.35%) and a positive correlation between knowledge and frequency [29]. In Uganda, only 27.3% practiced rational self-medication, with high antibiotic use common in developing countries [30]. In Saudi Arabia, 76.9% of students self-medicated, mostly using analgesics (84.9% vs. 46.9% in our study), with most feeling sufficiently knowledgeable and few reporting adverse effects, and 47.5% considered self-medication acceptable only after consulting a pharmacist [31]. Differences in antibiotic use between studies may reflect variations in legislation, education, and cultural practices.

The high use of analgesics and dietary supplements among respondents is consistent with data from Iran, where analgesics, antibiotics, and vitamins were commonly used [7]. Common motivations for self-medication—symptom recognition, confidence in knowledge, and avoiding waiting times—are also reported elsewhere. In Romania, Damian et al. found that 44% of antibiotic users had not consulted a doctor, reflecting a perception of self-diagnosis abilities among medically trained youth [8]. Instruction on self-medication risks was received by 64% of students, yet the practice remains prevalent, indicating that knowledge alone may not fully ensure cautious behavior [12]. In Alduraibi’s study, about 60% of students considered self-medication acceptable under controlled conditions, with confidence positively correlated with frequency [32]. In our study, perceived risks included drug interactions (27%), adverse reactions (24%), and masking of serious symptoms (19%), highlighting the need for continued awareness despite the perceived acceptability of self-medication. It is important to note that although students may be aware of the risks, this awareness does not appear to influence their self-medication behavior, as indicated by a very weak and non-significant correlation between confidence and frequency of self-medication (Spearman’s r = −0.041, *p* = 0.545). In our sample, students with higher self-reported confidence in their knowledge showed a tendency to engage less frequently in self-medication, suggesting a more selective use of OTC products rather than complete avoidance. However, the correlation is very weak, and these results should therefore be interpreted with caution. The lack of direct negative experiences, reported by 94% of respondents, may foster a false sense of security, as noted in previous studies [8]. Information sources play a key role: students primarily relied on the internet (32%), followed by university courses (27%) and pharmacists (19%). While specialists are trusted, heavy reliance on online sources raises concerns about information accuracy and potential misuse [33]. Encouragingly, over 71% of students support incorporating self-medication into the curriculum, reflecting awareness of current educational limitations. Dedicated modules on evaluating information sources, pharmacovigilance, and patient counseling could help promote responsible self-medication practices [34].

It is also notable that only 8.03% of students feel “very confident” in counseling patients about self-medication. This finding is crucial as it suggests a gap between theoretical knowledge and practical skills, which may negatively impact professional behavior. The moderate correlation between confidence in knowledge and self-perceived readiness for counseling (r = 0.45, *p* < 0.001) supports this hypothesis and highlights the need for targeted educational interventions, such as practical simulations and case studies. It is worth mentioning that respondents emphasized the necessity of filtering medical information and proposed solutions like developing a mobile app for information dissemination or using educational video materials. This initiative could serve as a starting point for creating scientifically validated digital resources useful both in university education and clinical practice.

A study published in 2020 reported a global prevalence of self-medication of 70.1% among students. Notably, the prevalence was significantly higher among medical students at 97.2%, compared to 44.7% in students from non-medical fields. This significant difference suggests that medical students are more prone to self-medication, possibly due to their access to medical knowledge and the perception of increased confidence in managing their own health [35]. A study conducted in Jordan found that medical students used analgesics more frequently (82.3%) compared to non-medical students (73%). Conversely, non-medical students reported higher usage of decongestants for cold symptoms (60.2% versus 47.6% among medical students). These differences may reflect variations in medication knowledge and perceptions of the risks associated with self-medication [36].

In general, individuals who perceive themselves as well-informed may make decisions more frequently, even without complete expertise. This phenomenon can be interpreted through the lens of the Dunning–Kruger effect, a cognitive bias well-documented in psychological literature, whereby individuals with limited experience may overestimate their knowledge [37,38]. Consequently, self-reported confidence in their own knowledge does not always equate to full medical expertise, highlighting the need for careful consideration when practicing self-medication [39]. Our data also emphasize that students with formal training in medicine or pharmacy may engage in self-medication, underlining the importance of vigilance, adherence to recommended guidelines, and awareness of potential errors such as incorrect dosing, inappropriate administration, or drug interactions [40,41].

In this context, professional training should include not only the transmission of knowledge but also the development of critical thinking, awareness of personal limitations, and ethical responsibility in relation to medical practice. Careful supervision of how students and young professionals apply their knowledge in practice is essential to prevent the internalization of risky behaviors and to support the development of correct and safe professional conduct. In the study by Hashemzaei et al., only 12.9% of students could correctly name two OTC drugs, highlighting potential risks related to dosage, suitability, and interactions. The study also showed that knowledge improves in higher-year students, justifying our focus on participants who have completed pharmacology courses. Pharmacy students demonstrated greater knowledge of self-medication, though their frequency of use was also higher, with no significant differences compared to medical students. In Saudi Arabia, only 13.7% of students consulted pharmacists, possibly reflecting confidence in their own knowledge. These findings emphasize that while students possess theoretical knowledge, practical experience remains essential for safe self-medication practices [29].

The practice of self-medication among medical and pharmacy students reflects not only individual behavior but also how they prepare for their future roles as responsible professionals. While common, self-medication can pose ethical challenges and affect professional judgment if used indiscriminately, as future physicians and pharmacists influence patient behavior and public health [34,42,43,44]. Studies indicate that students often have only partial awareness of risks such as drug interactions, incorrect dosages, or misdiagnosis [30,42]. Data on antibiotics show that some students use them without prescriptions, with varying perceptions of appropriateness [45,46]. Inappropriate self-medication can also impact the healthcare system through additional costs, hospitalizations, or ineffective treatments [47]. These findings highlight the need to strengthen the ethical dimension of education, ensuring students understand the responsibility associated with the safe and informed use of medications and the broader social consequences of their decisions.

Another essential element that emerges from the analysis of self-medication behaviors among Medicine and Pharmacy students is the need for a structured educational intervention by higher education institutions [7,48]. The faculty plays a crucial role not only in imparting theoretical knowledge but also in shaping professional attitudes and behaviors from the early years of training. There is a growing need to introduce dedicated modules focused on the responsible use of medications. Such courses should be interactive and integrated transdisciplinary, involving professors from pharmacology, bioethics, public health, and even medical psychology. It is not enough for students to understand how a medication works, it is essential that they also know when, why, and for whom it is appropriate. Thus, modern methods of improving skills and counseling methods are proposed that should be included in university programs, so as to reduce the gaps between theoretical and practical knowledge [49,50].

Moreover, fostering an educational environment that values caution, constructive skepticism, and the acknowledgment of personal limitations can help prevent the adoption of erroneous practices. In this regard, the faculty becomes not only a provider of knowledge but also a formative space for professional ethics.

However, the results of this study should be interpreted in light of certain methodological limitations. Firstly, the use of a cross-sectional design limits the ability to establish causal relationships between the analyzed variables. Self-medication behaviors may vary over time, and a longitudinal study would be more appropriate to assess these dynamics. Secondly, data were collected through self-reported questionnaires, which introduces the risk of bias, as respondents may be inclined to underreport the frequency of behaviors perceived negatively or overestimate their theoretical knowledge to conform to the ideal image of an informed student. Furthermore, the study was conducted at a single academic institution, limiting the generalizability of the findings at the national or international level, especially considering the cultural and institutional diversity present in health education systems. Another important limitation is the absence of a direct comparison with students from non-medical fields, which would have allowed for evaluating the specific impact of medical training on self-medication behaviors.

Despite these limitations, this study opens valuable directions for future research. A first step would be to expand the sample to include multiple universities, as well as students from other academic fields, with the aim of conducting a comparative analysis of self-medication practices between medical and non-medical students. Additionally, future research could employ a longitudinal design to track the evolution of attitudes and behaviors related to self-medication throughout the years of study. Another important area to explore is the qualitative analysis of the motivations behind self-medication, through interviews or focus groups, in order to gain an in-depth understanding of the psychological, cultural, and educational factors involved.

## 5. Conclusions

Self-medication remains highly prevalent among medical and pharmacy students, despite their theoretical training. Factors such as overconfidence in personal knowledge, easy access to online information, and the desire to avoid medical consultations contribute significantly to this behavior. To reduce instances of unsuitable or unsafe self-medication, university curricula should include dedicated, interactive, and interdisciplinary modules that promote not only knowledge but also critical thinking, professional responsibility, and practical skills. Future directions should focus on developing digital educational tools, clinical simulations, and integrated teaching strategies to support safe and informed decision-making from the early stages of medical training.

## Figures and Tables

**Table 1:** Sociodemographic data.

Characteristic	Value/Percent
Number of students	224
Females	71.42%
Youngest respondent	20 years
Oldest respondent	37 years
Age mean ± SD	22 ± 2 years
Most frequent age	22 years (36.16%)
Pharmacy students	20.08%

**Table 2:** Frequency and type of self-medication.

Frequency	Percent
Weekly	22.32%
Once a month	32.58%
Once in a few months	44.64%
Type of medicines	
Analgesics/antipyretics	46.87%
Supplements/vitamins	37.5%
Antihistamines	5.8%
Antibiotics	5.35%
Medicines for gastro-intestinal disorders	4.01%
Sedatives/hypnotics	0.44%
Reason	
Mild and recognizable symptoms	64.73%
Confidence in own knowledge	23.32%
Avoiding waiting time at the doctor’s office	5.8%
Recommendations from friends/family	5.35%
Accessibility to medicines	1.78%

**Table 3:** Perception about self-medication.

Risk	Percent
Drug interactions	26.78%
Adverse reactions	23.66%
Masking serious symptoms	18.75%
Wrong diagnostic	18.3%
Antibiotics resistance	11.6%
No risks	0.89%

**Table 4:** Sources of information.

Source of Information	Percent
Internet	32.14%
Books and lectures	27.23%
Pharmacist	19.19%
Family/friends	12.05%
Family doctor	9.37%

## Data Availability

The datasets that support the findings of this study are available from the first author upon reasonable request.

## Supplementary Materials

The following supporting information can be downloaded at: https://www.mdpi.com/article/10.3390/pharmacy13050127/s1, File S1: Questionnaire: Attitudes and behavior regarding self-medication.

## Abbreviations

The following abbreviations are used in this manuscript:

ANMDM	National Agency for Medicines and Medical Devices
OTC	Over the counter
