# Supplementary material for: Self-Medication: Attitudes and Behaviors Among Pharmacy and Medical Students
Source: Pharmacy (Basel). 2025 Sep 4;13(5):127. doi: 10.3390/pharmacy13050127 (PMC12452421; doi:10.3390/pharmacy13050127)
Supplement: Supplementary file 1 [file pharmacy-13-00127-s001.zip › pharmacy-3815292-supplementary.pdf]

# File S1: Questionnaire: Attitudes and behavior regarding self-medication

## 1. General information

1.1. **Age**: _______ years

1.2. **Genre**:

[ ] Male

[ ] Female

[ ] Other/ I would not like to say

1.3. **Academic specialization**:

[ ] Medicine

[ ] Pharmacy

1.4. **Study year**:

[ ] III (only for Medicine students)

[ ] IV (only for Pharmacy students)

## 2. Behaviors regarding self-medication

2.1. How frequently do you resort to self-medication?

[ ] Never

[ ] Once every few months

[ ] Approximately once a month

[ ] Weekly

[ ] Several times a week

2.2In the last 6 months, have you self-medicated with the following categories of medications?

[ ] Analgesics / Antipyretics
[ ] Antibiotics
[ ] Antihistamines
[ ] Gastrointestinal medication
[ ] Supplements / vitamins
[ ] Sedatives / anxiolytics
[ ] Other (specify): __________

2.3. What are the main reasons why you resort to self-medication?

[ ] Mild, easily recognizable symptoms
[ ] Avoiding waiting time at the doctor's office
[ ] Confidence in one's own knowledge after completing pharmacology courses
[ ] Accessibility to medicines
[ ] Recommendations from friends/family

# 3. Knowledge and perceived trust

3.1. How confident are you in your pharmacological knowledge when self-medicating? (1 = not at all confident, 5 = very confident)

1 2 3 4 5

3.2. You studied the risks of self-medication in university courses?

[ ] Yes

[ ] No

[ ] Don’t remeber

# 4. Perceptions and risks

4.1. Do you consider self-medication an acceptable practice under certain conditions?

[ ] Yes

[ ] No

[ ] Depends on the context

4.2. What are the most important risks associated with self-medication?

[ ] Adverse reactions
[ ] Drug interactions
[ ] Masking the symptoms of a serious illness
[ ] Antibiotics resistance
[ ] Wrong diagnotstic
[ ] I do not perceive significant risks

4.3. You have had a negative personal experience as a result of self-medication?

[ ] Yes

[ ] No
If yes, describe it in a few words: ___________________________

## 5. Sources of information used before self-medicating

[ ] Internet
[ ] Books / university lectures
[ ] Family doctor
[ ] Pharmacist
[ ] Family / friends
[ ] Other: _________

## 6. Exploratory components

6.1. Briefly describe a situation when self-medication was uself or problematic:

___________________________________________________________

6.2 What do you think could reduce inappropriate self-medication among young people

___________________________________________________________

**Do you think self-medication should be addressed separately in university curricula?**

[ ] Yes, mandatory

[ ] Yes, optionally

[ ] It’s not necessary

How prepared do you feel to counsel a patient about the risks of self-medication? (1 = not at all confident, 5 = very confident)
